# Changes in the Primary Metabolites of ‘Fengtang’ Plums during Storage Detected by Widely Targeted Metabolomics

**DOI:** 10.3390/foods11182830

**Published:** 2022-09-13

**Authors:** Xin Lin, Shian Huang, Qin Zhang, Shouliang Zhu, Xiaoqing Dong

**Affiliations:** 1 College of Agriculture, Guizhou University/Guizhou Engineering Research Center, Fruit Crops, Guiyang 550025, China; 2 Guizhou Workstation for Fruit and Vegetables, Guiyang 550025, China

**Keywords:** ‘Fengtang’ plum, primary metabolites, storage period, metabolism

## Abstract

Plums are one of the most popular stone fruits worldwide owing to their high nutritional value. After harvest, plum fruit quality and flavor change during storage; however, little is known about the changes in metabolites during this period. A comprehensive comparison of primary metabolites in ‘Fengtang’ plum fruits during storage is performed using widely targeted primary metabolomics. A total of 272 primary metabolites were identified by means of ultra-performance liquid chromatography and tandem mass spectrometry (UPLC-MS/MS) in the plums at different storage periods. There was a significant increase in the relative amounts of twenty-eight lipids, twenty amino acids and their derivatives, thirteen organic acids, ten saccharides and alcohols, six nucleotides and their derivatives, and two vitamins. A Kyoto Encyclopedia of Genes and Genomes (KEGG) enrichment analysis of differential metabolites revealed that glucosinolate biosynthesis, starch and sucrose metabolism, ascorbate and aldarate metabolism, lysine degradation, and other metabolic pathways were significantly enriched; therefore, changes in these metabolic pathways may be key to the quality and flavor change in ‘Fengtang’ plum fruits during storage. Our results provide a theoretical foundation and technical support to evaluate ‘Fengtang’ plum fruit quality.

## 1. Introduction

The plum (*Prunus salicina* L.) is a perennial drupe fruit in the genus *Prunus* of the Rosaceae family. Plum plantations and production in China rank first worldwide [1,2]. Plum fruits are rich in fiber, minerals, antioxidants, flavonoids, and glutathione, providing a rich source of nutrients for humans [1,3]. Plums are a typical respiration climacteric fruit that is harvested during high temperatures from July to August. However, plums exhibit vigorous postharvest physiological metabolic changes, resulting in a short storage period and shelf life, fast decay, and softening. Low-temperature storage is the most commonly used measure to delay fruit quality decline and reduce fruit decay loss, which can effectively inhibit physiological metabolic changes and prolong fruit life [3,4,5].

Primary metabolites, including sugars, amino acids, organic acids, and lipids, are essential nutrients in fruits that play an indispensable role in their growth, development, ripening, and senescence [6,7]. The composition and concentration of sugars and organic acids primarily determine fruit flavor [5,8], and the composition and abundance of amino acids affect fruit taste [9,10,11]. Although these studies have provided evidence that metabolic changes in plum fruits affect the fruit quality during storage, they have mainly focused on specific types of metabolites and offered only one perspective on the differences in quality. Consequently, to understand the contribution of different metabolites to the changes in plum quality during storage, it is necessary to conduct large-scale investigations to identify and quantify these metabolites.

Ultra-performance liquid chromatography and tandem mass spectrometry (UPLC-MS/MS)-based widely targeted metabolomics is a commonly used technique to identify and analyze metabolites [7,12,13,14]. It has several advantages, such as high throughput, rapid separation, high sensitivity, and comprehensive coverage. This technique has enabled the identification of the metabolites of strawberries, loquats, and passionflowers [7,13,14]. However, few studies have employed a variety of targeted metabolomic approaches to examine the components of plum fruits during low-temperature storage.

This study aims to investigate the changes in primary metabolites of ‘Fengtang’ plums during storage, using widely targeted metabolomics, by evaluating the dynamic changes in metabolite accumulation during UPLC-MS/MS analysis. A total of 272 primary metabolites, including 72 amino acids and their derivatives, 65 lipids, 52 organic acids, 37 nucleotides and their derivatives, 34 saccharides and alcohols, 12 vitamins, and 104 differential metabolites were detected in the plums at different storage periods. Compared with the single compound studied by predecessors [5,6,7,8,9,10,11], the primary metabolites of ‘Fengtang’ plum fruits during storage were comprehensively compared using the widely targeted primary metabolomics method. This study provides a further theoretical basis and reference for the qualitative detection and evaluation of the taste and quality of ‘Fengtang’ plum fruit.

## 2. Materials and Methods

### 2.1. Plant Materials

‘Fengtang’ plums, with a firmness of approximately 7.51–8.14 kg·cm^−2^, and total soluble solids (TSS) of approximately 12.5–14.0%, were collected from a farmer’s cooperative orchard in Zhenning County, Guizhou Province (105.52° E, 25.37° N) on 27 June 2020. Within four hours of harvest, the fruits were transported to the laboratory at the Department of Horticulture, College of Agronomy, Guizhou University. The fruits (containing 3 replicates, approximately 500 plums each) with uniform size and without insects or mechanical damage were selected for pre-cooling for 12 h and were then stored at 4 ± 0.5 °C. The physiological characteristics related to fruit quality were assessed using randomly selected fruits at 0, 10, 20, 30, 40, 50, and 60 d. The fruits stored for 0, 30, and 60 d were selected for widely targeted metabolomics analysis. All samples were promptly frozen in liquid nitrogen and stored in a −80 °C ultra-low temperature refrigerator (Thermo Fisher Scientific, Suzhou, China) for the UPLC-MS/MS analysis.

### 2.2. Measurements of Fruit Firmness, Respiration Rate, TSS, and Titratable Acid (TA)

The peels (approximately 1 mm thickness) of the plums were removed to measure the firmness of plum flesh at three different but equidistant peeling points on the equator of each fruit using a penetrometer (GY-4) with a probe diameter of 3.5 mm (three replicates of 50 plums). The results are presented in kg·cm^−2^. The respiration rate was measured by placing 10 fruits of uniform size in a 9.4 L desiccator containing a TEL7001 CO_2_ analyzer (ONSET, Bourne, MA, USA) for 1 h at 4 °C. The CO_2_ concentration was measured 3 times for 20 min, and the results are expressed as mg·kg^−1^·h. The TSS and TA were measured using a hand-held digital refractometer (Model PAL-BX/ACID1, Atago Co. Ltd., Tokyo, Japan) by dripping the fruit juice into the sample slot to determine the value of the TSS, followed by diluting the juice with distilled water for 50 times to calculate the value of the TA, values were expressed as Brix (percent) and percentage, respectively. The values of firmness, respiration rate, TSS, and TA were determined in triplicate.

### 2.3. Sample Preparation and Metabolite Extraction

After peeling the pericarp, the primary metabolites were extracted and analyzed by Wuhan Metware Biotechnology Co., Ltd. (Wuhan, China) in triplicate (in each group). The samples were freeze-dried in a vacuum freeze-dryer (Scientz-100F; Ningbo Scientz Biotechnology, Ningbo, China). The freeze-dried samples were crushed using a mixer mill (MM 400; Retsch GmbH, Haan, Germany) with zirconia beads for 1.5 min at 30 Hz. Lyophilized powder (100 mg) was dissolved in 1.2 mL 70% methanol and vortexed for 30 s every 30 min for 6 times in total, and the samples were placed in a refrigerator at 4 °C overnight. After centrifugation at 12,000 rpm for 10 min, the extracts were filtered (SCAA-104, 0.22 μm pore size; ANPEL, Shanghai, China) before the UPLC-MS/MS analysis.

### 2.4. UPLC Conditions

A UPLC ESI-MS/MS system (UPLC, SHIMADZU Nexera X2, Shimadzu, Kyoto, Japan; MS, 4500Q TRAP, Applied Biosystems, Waltham, MA, USA) was used for sample extraction. The analytical conditions were as follows, UPLC: column, Agilent SB-C18 (1.8 µm, 2.1 mm × 100 mm). The mobile phase consisted of solvent A, pure water with 0.1% formic acid, and solvent B, acetonitrile with 0.1% formic acid. Sample measurements were performed with a gradient program that employed the starting conditions of 95% A, 5% B. Within 9 min, a linear gradient to 5% A 95% B was programmed, and a composition of 5% A, 95% B was kept for 1 min. Subsequently, a composition of 95% A, 5.0% B was adjusted within 1.10 min and kept for 2.9 min. The flow velocity was set at 0.35 mL per minute. The column oven was set to 40 °C. The injection volume was 4 μL. The effluent was alternatively connected to an ESI-triple quadrupole-linear ion trap (Q TRAP)-MS.

### 2.5. ESI-Q TRAP-MS/MS

LIT and triple quadrupole (QQQ) scans were acquired on a triple quadrupole-linear ion trap mass spectrometer (Q TRAP), AB4500 Q TRAP UPLC/MS/MS System, equipped with an ESI Turbo Ion-Spray interface, operating in positive and negative ion modes and controlled by Analyst 1.6.3 software (AB Sciex, Toronto, ON, Canada). The ESI source operation parameters were as follows: ion source, turbo spray; source temperature 550 °C; ion spray voltage (IS) 5500 V (positive ion mode)/−4500 V (negative ion mode); ion source gas I (GSI), gas II (GSII), and curtain gas (CUR) were set at 50, 60, and 25.0 psi, respectively; the collision-activated dissociation (CAD) was high. Instrument tuning and mass calibration were performed with 10 and 100 μmol/L polypropylene glycol solutions in QQQ and LIT modes, respectively. QQQ scans were acquired as MRM experiments with collision gas (nitrogen) set to medium. DP and CE for individual MRM transitions were done with further DP and CE optimization. A specific set of MRM transitions were monitored for each period according to the metabolites eluted within this period.

### 2.6. Metabolite Identification and Quantification

For the qualitative analysis of metabolites, the primary and secondary MS data were used to annotate metabolites based on the self-built metware database (MWDB) (Wuhan Metware Biotechnology Co., Ltd., Wuhan, China) (http://www.metware.cn/, accessed on 1 December 2020) and public metabolite databases (MassBank (http://www.massbank.jp), KNAPSAcK (http://kanaya.naist.jp/KNApSAcK, accessed on 1 December 2020), Human Metabolome Database (HMDB; http://www.hmdb.ca, accessed on 1 December 2020), MoTo DB (http://www.ab.wur.nl/moto, accessed on 1 December 2020), and METLIN (http://metlin.scripps.edu/index.php, accessed on 1 December 2020)). During the analysis, the isotope signals–the repeated signals containing K^+^, Na^+^, and NH_4_^+^ ions–and the repeated signals of fragment ions that are other substances with larger molecular weight were removed. The quantification of metabolites was performed by multiple MRM analyses using triple quadrupole mass spectrometry.

In the MRM mode, the quadrupole first selected the precursor ions of the target substance, and excluded ions corresponding to other molecular weight substances to preliminarily eliminate interference. After the precursor ions were induced to ionize in the collision chamber, they broke into many fragment ions. The fragment ions were filtered by QQQ to select a required characteristic of fragment ion, to eliminate the interference of non-target ions so that the quantification was more accurate and repeatability, better. If the compound had the same precursor ion, qualifier, and quantifier at the same retention time (RT), the qualitative process had two situations: If the secondary spectra (Q3/Q4/Q5/Q6/Q7) and other information of the two metabolites were consistent, they belonged to an isomer at the mass spectrum level. Mass spectrometry cannot distinguish isomers. If the secondary spectrum information of the two metabolites was different, we chose the differential ion as the characteristic ion and its quantitative ion.

Mixtures of the QC samples were prepared by mixing equal amounts of plum fruit from different storage periods. The repeatability of the samples used for the analysis was determined using the same treatment method. One QC sample was inserted in every ten samples during the instrumental analysis to ensure that the analysis procedure was repeatable.

### 2.7. Statistical Analysis

The mass spectrometry data were processed using Analyst 1.6.3. The metabolites were measured by the multiple reaction monitoring mode (MRM) analysis of triple quadrupole mass spectrometry, based on secondary spectrum information from the MWDB (metware database), which is a public database of metabolic information produced by Wuhan Metware Biotechnology Co. Ltd. A multivariate statistical analysis was used to perform a principal component analysis (PCA) of the samples [14,15,16]. The orthogonal projections to latent structures discriminant analysis (OPLS-DA) model was used to analyze the metabolomic data [17]. The PCA and OPLS-DA were performed using the R package (www.r-project.org, accessed on 1 December 2020). PCA was used to recombine the original variables into new, mutually independent variables through orthogonal transformations.

The OPLS-DA and PCA were conducted to analyze and verify the differences and reliability of metabolites in the samples. Differential metabolites were calculated by combining *p*-values (*t*-test) or fold changes of the univariate analysis with VIP (variable importance plot) scores for the OPLS-DA model. Metabolites with VIP ≥ 1 and fold changes ≥ 2 or fold changes ≤ 0.5 were considered differential metabolites for group discrimination. The Kyoto Encyclopedia of Genes and Genomes (KEGG) functional annotation and pathway enrichment analysis of the differential metabolites was performed to identify enriched metabolic pathways.

## 3. Results

### 3.1. Changes in Firmness, Respiration Rate, TSS Content, and TA Content in ‘Fengtang’ Plum Fruits

**Figure 1:**
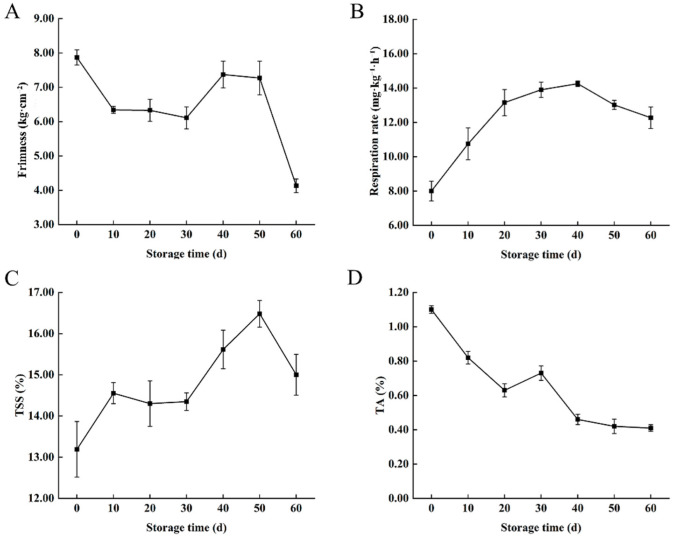
Physiological characteristics of ‘Fengtang’ plum fruits: (**A**) Firmness, (**B**) Respiration rate, (**C**) TSS, and (**D**) TA.

Firmness, respiration rate, TSS, and TA content are essential indicators of fruit quality [18]. In the present study, the fruit firmness gradually decreased during storage. The firmness of the fruit was 7.87 kg·cm^−2^ at 0 d, which was reduced to 4.13 kg·cm^−2^ at 60 d (Figure 1A). The respiration rate increased significantly during storage, reaching a maximum of 14.25 mg·kg^−1^·h^−1^ on 40 d, which then decreased gradually (Figure 1B). The TSS content also increased during storage from 13.19% on 0 d to 15.00% on 60 d (Figure 1C). In contrast, the TA content decreased significantly during storage, from 1.1% on d 0 to 0.41% on 60 d (Figure 1D). Moreover, the differences in the indexes indicated that there were differences in metabolites during storage.

### 3.2. Analysis of the Primary Metabolites in ‘Fengtang’ Plum Fruits

A total of 272 primary metabolites were detected using UPLC-MS/MS, including 72 amino acids and their derivatives, 65 lipids, 52 organic acids, 37 nucleotides and their derivatives, 34 saccharides and alcohols, and 12 vitamins (Table S1).

**Figure 2:**
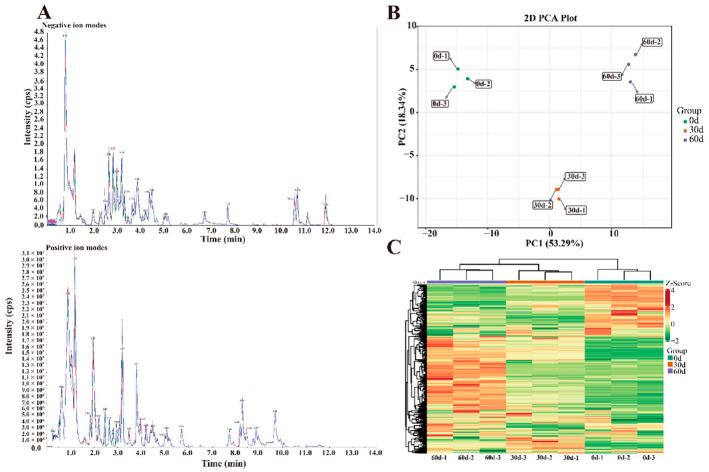
Differences in ‘Fengtang’ plum fruit samples at different storage periods: (**A**) TIC overlap map detected by mass spectrometry (MS), (**B**) PCA score map, and (**C**) Cluster thermogram of primary metabolites.

The total ion flow diagram (TIC) of the essential control of the plum sample in positive and negative ion modes overlapped (Figure 2A). The results show that the peaks and retention times of each sample overlapped, indicating that the systematic error of the experimental instrument is within the controllable range and that further analysis of the metabolomic data is reliable. In addition, 272 primary metabolites were analyzed using PCA. PC1 and PC2 were 53.29% and 18.34%, respectively, and the contribution of the two principal components was 71.63%; this suggests that the representative properties of approximately 71.63% of the metabolites were well aggregated, according to the 2D PCA scatter plots in Figure 2B. The QC samples were mixed, and the separation trend of metabolites among the three groups (0-, 30-, and 60-d) was apparent. The samples in each group were not separated, and the sample data were reliable to be used for subsequent analysis of differential metabolites.

The primary metabolites of ‘Fengtang’ plum fruits were studied using a clustered heatmap, and variations in the primary metabolites over time. The results revealed differential primary metabolites in the fruits during the different storage periods, most of which showed an increasing trend (Figure 2C). Furthermore, the accumulation of primary metabolites in fruits may influence fruit quality and flavor.

### 3.3. Screening of Differential Metabolites in ‘Fengtang’ Plum Fruits

To identify the differential metabolites in ‘Fengtang’ plum fruits at different storage periods, the differential metabolites among the groups were screened according to the following criteria: VIP ≥ 1, fold changes ≥ 2, fold changes ≤ 0.5, and *p* < 0.05 (for *t*-test).

**Figure 3:**
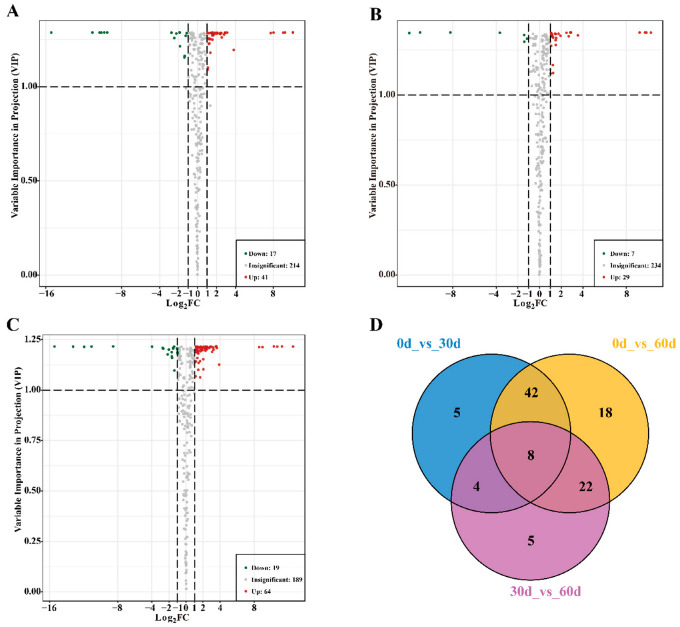
Screening of differential metabolites in ‘Fengtang’ plum fruits during storage: (**A**) Volcanic maps of differential metabolites at 0 d vs. 30 d, (**B**) Volcanic maps of differential metabolites at 30 d vs. 60 d, (**C**) Volcanic maps of differential metabolites at 0 d vs. 60 d. (**D**) Venn diagram of differential metabolites between groups.

There were 58 differential metabolites between the 0- and 30-day groups; 41 were upregulated and 17 were downregulated. There were 36 differential metabolites between the 30- and 60-day groups; 29 were upregulated and 7 were downregulated. Moreover, 83 differential metabolites were identified between the 0- and 60-day groups; 64 were upregulated and 19 were downregulated (Figure 3A–C). The Venn diagram of the differential metabolites among the groups is shown in Figure 3D. The diagram shows eight differential metabolites, including four amino acids and derivatives (L-methionine, L-lysine-butanoic acid, *N*-acetyl-L-tryptophan, and S-(5′-adenosy)-L-homocysteine), two lipids (LysoPC 15:1, and LY16:2), pyridoxal, and L-(sn-glycero-3-phosphoserine) 1D-myo-inositol. These eight differential metabolites accumulated during storage (Table S2). Moreover, different metabolite profiles may be the main reason for the varying fruit quality and flavors in the course of storage.

### 3.4. Differential Accumulation of Amino Acids and Their Derivatives in ‘Fengtang’ Plum Fruits during Storage

**Figure 4:**
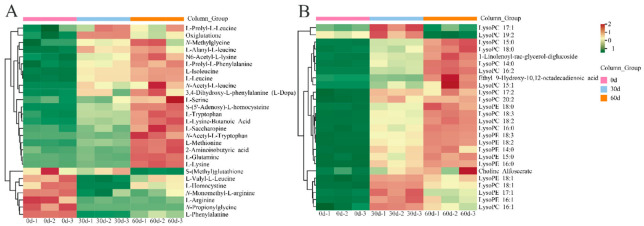
Heatmap of differential metabolites during storage of ‘Fengtang’ plum fruits: (**A**) Heatmap of amino acids and derivatives, and (**B**) Heatmap of lipids.

The relative amounts of amino acids and their derivatives in ‘Fengtang’ plum fruits were determined to study the variations in amino acids and derivatives over storage duration. We detected 72 amino acids and their derivatives, of which 27 were differential metabolites (Table S2). Figure 4A shows a heatmap of the accumulation of amino acids and their derivatives in ‘Fengtang’ plum fruits during different storage periods. These findings indicate that most amino acids and their derivatives increase with storage duration. The levels of the seven amino acids and their derivatives (S-(methyl)glutathione, L-Valyl-L-leucine, L-homocysteine, *N*-monomethyl-L-arginine, L-arginine, *N*-propionylglycine, and L-phenylalanine) were higher at 0 d than at 30 and 60 d. The relative contents of L-prolyl-L-leucine and glutathione in ‘Fengtang’ plum fruits at 30 d were higher than those at 0 and 60 d. The other 18 amino acids and their derivatives accumulated during storage. Therefore, the accumulation of different amino acids and their derivatives leads to varying fruit quality in the course of storage.

### 3.5. Differential Accumulation of Lipids in ‘Fengtang’ Plum Fruits during Storage

As shown in Figure 4B, a total of 26 differentially accumulated lipids were screened, out of which 24 lipids accumulated during storage; however, the relative contents of these lipids were not significantly different between the 30- and 60-day groups (Table S2). The relative contents of two lipids, LysoPC 17:1 and LysoPC 19:2, in ‘Fengtang’ plum fruits at 60 d were lower than those at 0 and 30 d.

### 3.6. Differential Accumulation of Organic Acids, Saccharides and Alcohols, Nucleotides and Their Derivatives, and Vitamins in ‘Fengtang’ Plum Fruits during Storage

**Figure 5:**
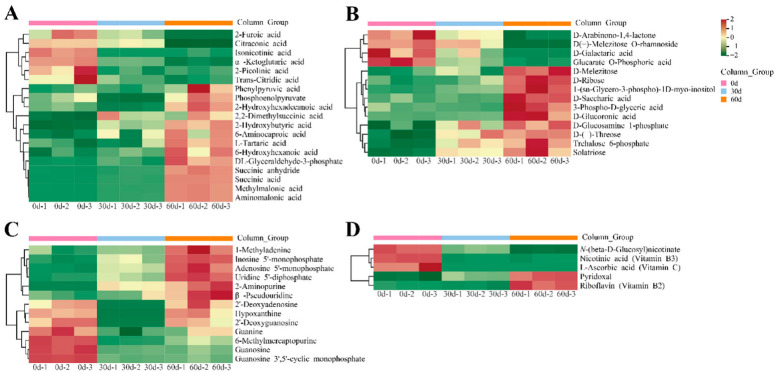
Heatmap of differential metabolites during storage of ‘Fengtang’ plum fruits: (**A**) Heatmap of organic acids, (**B**) Heatmap of saccharides and alcohols, (**C**) Heatmap of nucleotides and derivatives, and (**D**) Heatmap of vitamins.

Among the identified differential metabolites, 19 organic acids, including 13 organic acids such as succinic anhydride, succinic acid, methylmalonic acid, aminomalonic acid, L-tartaric acid, and DL-glyceraldehyde 3-phosphate acid were significantly accumulated on 60 d (Figure 5A).

Four saccharides and alcohols (D-arabinono-1,4-lactone, D-galactaric acid, glucarate O-phosphoric acid, and D(+)-melezitose O-rhamnoside) were significantly higher at 0 d than at other storage periods. The amounts of ten saccharides and alcohols, such as D-ribose, solatriose, and D-melezitose accumulated during storage (Figure 5B).

During storage, the differential metabolites of ‘Fengtang’ plum fruits included thirteen nucleotides and their derivatives as well as five vitamins (Figure 5C,D). The relative concentrations of nicotinic, L-ascorbic acid, and *N*-(β-D-glucosyl)nicotinate were significantly higher at 0 d than at 30 and 60 d, whereas the contents of pyridoxal and riboflavin were significantly lower (Table S2). Moreover, the different metabolite profiles may be the main reasons for the varying fruit quality and flavor in the course of storage.

### 3.7. KEGG Functional Annotation and Enrichment Analysis of the Differential Metabolites in ‘Fengtang’ Plum Fruits

**Figure 6:**
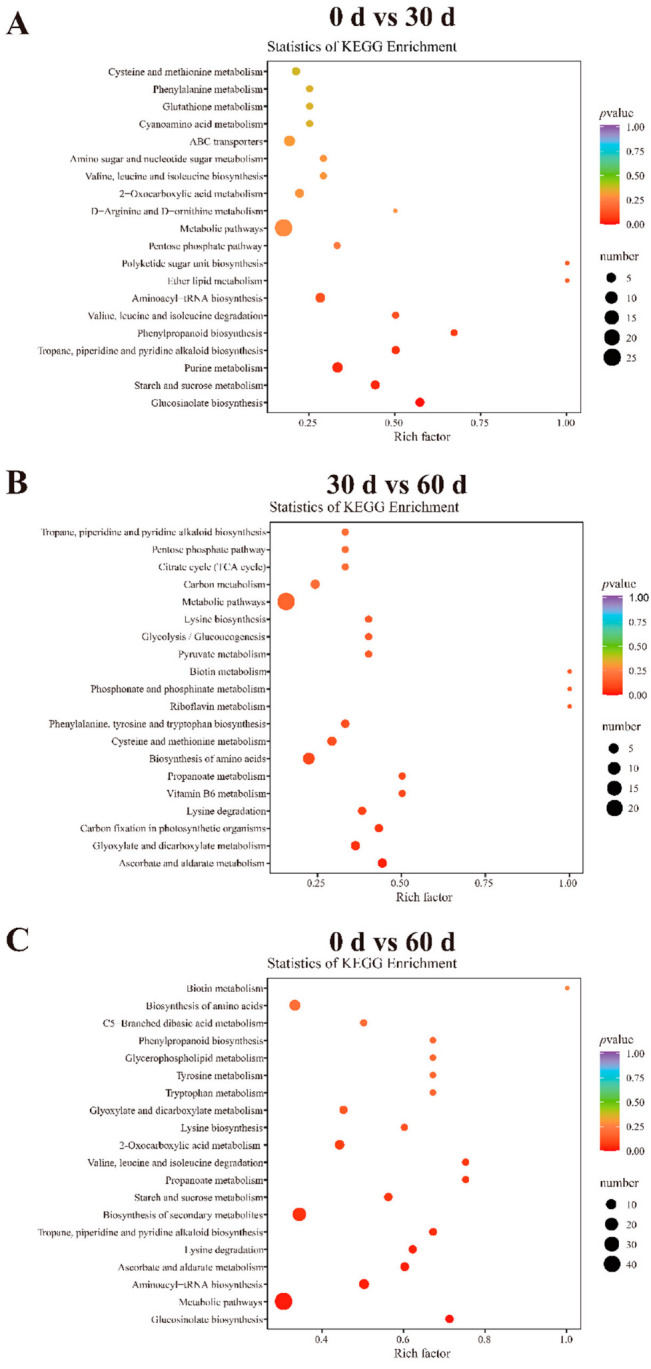
KEGG enrichment in ‘Fengtang’ plum fruits at different storage periods: (**A**) 0 d vs. 30 d, (**B**) 30 d vs. 60 d, and (**C**) 0 d vs. 60 d.

To understand the changes in the metabolism of the differential metabolites in ‘Fengtang’ plum fruits during storage, a KEGG functional annotation and pathway enrichment analysis of the differential metabolites were performed. The analysis revealed that of the 58 differential metabolites between the 0- and 30-day groups, 21 were annotated in KEGG and 34 to metabolic pathways; glucosinolate biosynthesis, starch and sucrose metabolism, purine metabolism, and scopolamine piperidine and pyridine alkaloid biosynthesis were significantly enriched (*p* < 0.05, Figure 6A). The 36 differential metabolites between the 30- and 60-day groups were annotated to 50 metabolic pathways, and only two metabolic pathways, ascorbate and aldarate metabolism and glyoxylate and dicarboxylate metabolism, were significantly enriched (*p* < 0.05, Figure 6B). Out of the 83 differential metabolites between the 0- and 60-day groups, 59 were annotated to metabolic pathways. Among these, seven metabolic pathways were significantly enriched: glucosinolate biosynthesis, metabolic pathways, biosynthesis of cofactors, aminoacyl-tRNA biosynthesis, ascorbate, and aldarate metabolism, lysine degradation, and tropane piperidine and pyridine alkaloid biosynthesis (Figure 6C). Consequently, the different metabolic pathways could be another major reason for the varying fruit quality and flavor during storage.

### 3.8. Correlation Analysis between Primary Metabolites and Fruit Quality in ‘Fengtang’ Plum Fruits

**Figure 7:**
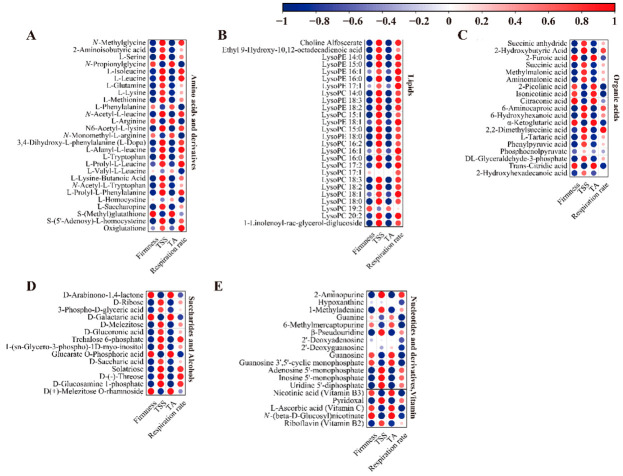
The correlation analysis between physiological characteristics and differential metabolites in ‘Fengtang’ plum fruits: (**A**) Amino acids and derivatives, (**B**) Lipids, (**C**) Organic acids, (**D**) Saccharides and alcohols, and (**E**) Nucleotides and derivatives, vitamins.

The relationship between differential metabolites and quality indices was evaluated through correlation analysis; one hundred and four differential metabolites and four quality indices were studied (Figure 7). The results showed that most amino acids and their derivatives were negatively associated with firmness and TA content, and positively associated with the TSS and respiration rate. In contrast, seven amino acids and derivatives (*N*-propionylglycine, L-phenylalanine, L-arginine, *N*-monomethyl-L-arginine, L-valyl-L-leucine, L-homocysteine, and S-(methyl)glutathione) were positively associated with firmness and TA content and negatively associated with the TSS content and respiration rate (Figure 7A).

In addition to LysoPC 19:2, other lipid metabolites were negatively associated with firmness and TA content and positively associated with respiration rate; moreover, all lipids were positively associated with the TSS content (Figure 7B).

Among the nineteen organic acid metabolites, six organic acid metabolites were positively associated with firmness and TA content and negatively associated with the TSS content and respiratory rate. Conversely, the other 13 organic acids were negatively associated with firmness and TA content and positively associated with the TSS content and respiration rate (Figure 7C).

Four saccharides and alcohols (D-arabinono-1,4-lactone, D-galactaric acid, glucarate-o-phosphoric acid, and D(+)-melezitose-o-rhamnoside) were positively associated with firmness and TA content and negatively associated with the TSS content and respiratory rate (Figure 7D). Among other differential metabolites, some were negatively associated with firmness and TA content. Moreover, nicotinic acid and L-ascorbic acid were positively associated with firmness and TA content (Figure 7E). Consequently, differences in the contents of metabolites associated with firmness, respiration rate, the TSS, and TA during storage could be another key reason for differences in fruit quality and flavor.

## 4. Discussion

Firmness, respiration rate, TSS, and TA content are essential indicators of fruit quality [18,19]. The firmness in plum fruit at 40 and 50 d was higher than that in fruit at 30 d, which may be caused by low-temperature stress. Low-temperature stress affects the metabolism of the fruit cell wall substances, resulting in fruit failure to soften normally as well as the fruit being spongy or lignified [20,21]. However, the fruit senescence at the later stage of storage makes fruit firmness drop sharply. Primary metabolites such as sugars, organic acids, and amino acids are essential nutrients in fruits. During storage, the contents of these metabolites in plums vary, affecting the flavor and quality of the fruits. Previous studies on the changes in the metabolites of plum fruits during storage have focused on one or more specific metabolites such as sugars (glucose, fructose, sorbitol, and sucrose) [8,22], organic acids (malic, citric, succinic, and oxalic acids) [5,23], and amino and ascorbic acids [24,25]. However, the metabolomic differences in ‘Fengtang’ plum fruits during storage have not been reported. In this study, UPLC-MS/MS was used to analyze the quality and flavor changes of ‘Fengtang’ plum fruits during storage, and six classes of three-hundred and seven primary metabolites were detected. The primary metabolites were divided into amino acids and their derivatives, nucleotides and their derivatives, lipids, organic acids, saccharides, alcohols, and vitamins. These metabolites are involved in several metabolic pathways, such as glycometabolism, amino acid metabolism, the pentose phosphate pathway, the glycolytic process, the tricarboxylic acid cycle, and the glyoxylate cycle. Therefore, this study reveals the differences in quality and taste of ‘Fengtang’ plum fruits during different storage periods on a metabolic level.

The content and composition of sugars and acids in the fruit can be used as an essential index to evaluate fruit flavor [2]; the sugar-to-acid ratio is usually used to assess the fruit’s taste and quality [26,27]. In this study, the TSS content gradually increased during storage, whereas the TA content steadily declined; this result was consistent with Wang et al. [5] and Xu et al. [8]. In our study, 34 saccharides and alcohols were detected in ‘Fengtang’ plum fruits during storage, of which 14 considerably differed during storage time. Moreover, D-fructose, D-glucose, D-mannose, and D-galactose were the most abundant (Table S1), which is consistent with previous studies [5,11,28,29,30]. In addition, the relative contents of solatriose, D-(−)-Threose, and D-glucosamine 1-phosphate at 0 d were significantly lower than those at 30 and 60 d, which may partly explain the increased sweetness of the fruit during storage. Therefore, the flavor of the ‘Fengtang’ plum fruits at 0 d was worse than those at other periods.

Furthermore, L-malic acid, isocitrate, citric acid, succinic acid, succinic anhydride, and L-tartaric acid were the most abundant organic acids (Table S1). A total of 52 organic acids were detected; the amounts of 19 of these organic acids significantly changed during storage, and 13 significantly accumulated during storage. Previous studies found that the taste of plum fruits changed dramatically during storage, in which soluble solids accumulated and the content of organic acids decreased [5,31]. The relative contents of succinic anhydride, succinic acid, and L-tartaric acid on 0 d were substantially lower than those at 30 and 60 d; however, the relative amounts of L-malic acid and isocitrate declined during storage. Therefore, the acidic taste of the fruit decreased during storage, which made the flavor and quality of the fruit better than at 0 d of storage.

The type and content of amino acids, including glutamic acid, aspartic acid, and lysine are among the leading indicators for evaluating fruit taste. Alanine, glycine, serine, and proline are sweet amino acids. Thyroid acids, L-cysteine, and L-phenylalanine are aromatic amino acids. Arginine, valine, leucine, L-isoleucine, and tryptophan are bitter amino acids [32]. Previous studies have identified 15–17 amino acids in plum fruits [11,33]. In this study, 72 amino acids and their derivatives were detected in ‘Fengtang’ plum fruits, and the amounts of 27 of the 72 amino acids were markedly changed during storage. The two fresh and sweet amino acids, L-serine and L-lysine, increased significantly during storage, which increased the sweet taste of the fruit; however, the levels of L-phenylalanine, L-arginine, and other bitter amino acids decreased during storage. The changes in these metabolites made the fruits of 30 and 60 d better than those of 0 d in flavor.

In addition, 65 lipids, 37 nucleotides and derivatives, and 12 vitamins were detected during storage. The amounts of twenty-six lipids, thirteen nucleotides and their derivatives, and five vitamins changed significantly during storage. Lipids are the main volatile organic compounds in ‘Fengtang’ plum fruits and the precursor metabolite of esters. During storage, many lipids accumulate in ‘Fengtang’ plum fruits, thereby increasing the concentrations of volatile substances. Lipids are precursors of flavor substances, and vitamins have antioxidant effects. Therefore, significant changes in these substances during storage also lead to changes in fruit quality. Moreover, the KEGG enrichment analysis revealed that glucosinolate biosynthesis, starch and sucrose metabolism, ascorbate and aldarate metabolism, lysine degradation, and other metabolic pathways were significantly enriched in stored ‘Fengtang’ plum fruits. The number of metabolites in these metabolic pathways changed significantly during storage, potentially leading to differences in flavor and quality of the ‘Fengtang’ plum fruits. The results of the present study provide a theoretical basis and reference for the qualitative detection and evaluation of the taste and quality in ‘Fengtang’ plum fruits.

A key concern with regard to the findings of the present study is that the accumulation of metabolites, including primary metabolites and secondary metabolites, influences fruit quality and flavor. Primary metabolites are essential for maintaining vital cell activities and are the precursor substances of secondary metabolites, such as sugars and amino acids. Therefore, further studies are required to investigate the effects of metabolite accumulation on fruit quality and flavor.

## 5. Conclusions

In this study, widely targeted metabolomics (UPLC-MS/MS) was used to identify and screen the primary metabolites in ‘Fengtang’ plum fruits during different storage periods. This work provides comprehensive information on both metabolite compositions and abundances in plum, an important commercial fruit. In this study, 104 differential metabolites made the fruit quality and flavor better at 30 and 60 d than at 0 d. The fruit of 60 d had chilling injury and senescence, and the fruit firmness of 60 d was lower than that of 30 d. In conclusion, the fruit quality after 30 d was better than that following 0 and 60 d of storage. The changes in primary metabolites may be the fundamental reason for the differences in fruit flavor and quality in ‘Fengtang’ plum fruits during storage. These findings provide insight into the improvement of the theoretical basis and reference for the qualitative detection and evaluation of taste and quality in ‘Fengtang’ plum fruit. However, further molecular evidence, to support the effect of differential metabolites on fruit quality and flavor, needs to be confirmed in the near future.

## Data Availability

Data are presented within the article.

## Supplementary Materials

The following supporting information can be downloaded at: https://www.mdpi.com/article/10.3390/foods11182830/s1, Table S1: Primary metabolites at different storage periods in ‘Fengtang’ plum fruits by UPLC-MS/MS. Table S2: Differential metabolites in ‘Fengtang’ plum fruits during storage.
